# A Randomized Crossover Trial on the Acute Cardiovascular Demands During Flywheel Exercise

**DOI:** 10.3389/fphys.2021.665462

**Published:** 2021-06-25

**Authors:** Damir Zubac, Vladimir Ivančev, Zoran Valić, Rado Pišot, Cécil J. W. Meulenberg, Irhad Trozić, Nandu Goswami, Boštjan Šimunič

**Affiliations:** ^1^Science and Research Center Koper, Institute for Kinesiology Research, Koper, Slovenia; ^2^Faculty of Kinesiology, University of Split, Split, Croatia; ^3^Department of Integrative Physiology, School of Medicine, University of Split, Split, Croatia; ^4^Gravitational Physiology, Aging and Medicine Research Unit, Physiology Division, Otto Loewi Center of Vascular Biology, Immunity and Inflammation, Medical University of Graz, Graz, Austria

**Keywords:** yo-yo exercise, oxygen uptake, muscle loading, blood pressure monitoring, flow mediate dilation

## Abstract

In a randomized crossover trial, we examined whether age plays a role in the mean arterial pressure (MAP) response during a vigorous flywheel exercise of varying load. We hypothesized that the magnitude of increase in the MAP during the flywheel exercise would increase in proportion to advancing age, thereby imposing a significant challenge to the cardiovascular system. A total of 30 participants of both sexes (age range from 20–55 y, 37% women) underwent a detailed medical examination, and their maximal oxygen uptake was determined. They performed a squat exercise (2 sets × 7 repetitions) on a flywheel ergometer at three randomly assigned moments of inertia set at 0.025, 0.05, and 0.075 kg m^2^, while the cardiovascular response was continuously recorded via a Task force monitor. Compared to the resting values, robust rises in the MAP were observed during all three flywheel loads, reaching the highest value of 179 ± 4 mmHg (*p* = 0.001) during the highest load. In parallel, the cardiac index (cardiac output normalized by the body surface area) was two-fold greater during all the flywheel loads compared to rest, and at a high load, exclusively, the total peripheral resistance increased by 11% (*p* = 0.001). The rise in heart rate compensated for a load-dependent drop in the stroke index (stroke volume normalized by the body surface area). In our study population, no correlations were observed between the relative increase in the MAP and the participants’ age for the three flywheel loads. The present findings suggest that the larger moments of inertia impose a substantial burden to the cardiovascular system, without apparent associated age-differences of the relative magnitude of MAP rise throughout the exercise.

## Introduction

To counter muscle atrophy, an emerging public health concern associated with aging and sedentary lifestyle trends, exercise scientists have suggested the gravity-independent flywheel ergometer as one of the most effective resistance exercise model (Tesch et al., 2004, 2016; Seynnes et al., 2007; Norrbrand et al., 2010; Lundberg et al., 2019). A greater muscle activation, likely due to the brief episode of skeletal muscle overload during the eccentric phase of movement, was reported in the literature using the flywheel rotating wheel when compared to a classical resistance exercise (Norrbrand et al., 2010). Different experimental designs, including randomized control trials in the general population (Seynnes et al., 2007; Lundberg et al., 2019), bed rest studies (Tesch et al., 2004), unilateral lower limb suspension models (Tesch et al., 2016), and rehabilitation programs (Fernandez-Gonzalo et al., 2014), have all outlined the positive effects of a flywheel-based exercise on the skeletal muscle mass, architecture, neural activation, balance, and power output. Even three weeks of flywheel training provided an adequate loading stimulus that augmented muscle architecture remodeling (a ∼6% increase in muscle cross sectional area, ∼10% increase of muscle fiber pennation angle, and ∼8% increase in muscle fiber fascicle length), in parallel to ∼30% enhance in the muscle power output and ∼20% increase of electromyography (EMG) activity of the quadriceps femoris muscle (Seynnes et al., 2007).

In spite of the growing interest of recreationally active individuals (including seniors) using the flywheel resistance exercise routine (Tesch et al., 2017), a limited number of investigations had focused on the cardiovascular response to a whole-body resistance exercise on a flywheel ergometer at any inertial load. Apparently, researchers typically overlook to quantify the cardiovascular burden, imposed by the vigorous muscle loading exercise. In fact, it is still unknown whether this type of resistance exercise imposes an additional challenge to the cardiovascular system, potentially due to the large contractile work imposed by the flywheel exercise during the entire range of motion, thereby creating an additional mechanical occlusion of large blood vessels and leading to a potentially greater burden to the cardiovascular system. Taken together, the cardiovascular response to the flywheel exercise is still unknown, and to promote lifestyle modifications or optimize rehabilitation interventions via the flywheel exercise prescription, the absence of knowledge on the response currently precludes a safer exercise optimization for different populations.

Currently, there are no established guidelines concerning the criteria for a hypertensive response threshold during resistance testing, even though a multiple joint whole-body resistance exercise, such as squat is generally recommended by the literature (Garber et al., 2011), primarily due to the large muscle mass activation. The American Heart Association (AHA) position stand (Fletcher et al., 2013) as advocates for a mandatory exercise capacity testing in the middle-aged population (>45 years of age) prior to engaging in a vigorous exercise program. Exercise test guidelines are limited to the Bruce treadmill or cycle-ergometers tests, performed to maximally stress and provoke the cardiovascular response during exercise (Pescatello et al., 2004; Fletcher et al., 2013). Earlier studies looking at the blood pressure (BP) response during human movement were predominantly focused on orthostatic tolerance tests (Goswami et al., 2015), aerobic exercise interventions (Mora-Rodriguez et al., 2018), or isolated submaximal lower-limb contractions (Lamotte et al., 2010; Massaferri et al., 2015; Trinity et al., 2018). A study by Lovell et al. (2011) observed a substantial increase in the BP during a classical squat-exercise in sedentary male seniors. In that study, the BP response was assessed via a photoplethysmography-based Finometer, and showed a robust increase in the BP, with average readings during a squat exercise reaching as high as 231/128 mmHg. However, from the latter study, it is not clear whether this sharp, transient rise in the BP response is due to a sedentary lifestyle or ageing, *per se*, since it is well-known that the resting BP increases with age (Pescatello et al., 2004; Garber et al., 2011) and is modified via physical activity levels across the lifespan (Arbab-Zadeh et al., 2004). To answer this question, we recently investigated the effects of an acute flywheel exercise on the vascular endothelium function (measured via flow-mediated dilation, FMD technique) and BP response in healthy men of varying age (between 19 and 57 y), and their cardiorespiratory fitness (Zubac et al., 2021a). We found that an acute flywheel exercise, set at 0.075 kg m^2^, triggers an excessive and transient BP rise (with average readings reaching ∼230/115 mmHg), but does not impair the FMD function among the study participants. The key finding of that study was that healthy individuals as they get older and adopt a sedentary lifestyle and have a lower V̇O_2_ max. tend to have lower FMD capacity, indicating an impaired vascular health. However, the latter findings were limited to healthy men of varying cardiorespiratory fitness and age, while a recent systematic review on the effects of flywheel exercise on strength-related variables clearly indicates that there is a lack of randomized controlled trials among the female population taking into consideration their cycle phase and subsequent influence of hormonal fluctuations on performance outcomes (González et al., 2021). Therefore, the purpose of this study was to investigate the effects of the squat exercise performance through a flywheel ergometer on the physiological response in healthy, active participants of both sexes across a wide age-range. We reasoned that a long-lasting physical exercise involvement (and subsequently, a higher V̇O_2_ max.) would not protect against the age-dependent cardiovascular response to flywheel exercise. Collectively, this study aimed to examine whether age plays a role in the mean arterial pressure (MAP) response during a vigorous flywheel exercise of varying load. We hypothesized that the magnitude of increase in the MAP during the flywheel exercise would increase in proportion to advancing age, thereby imposing a significant challenge to the cardiovascular system.

## Materials and Methods

### Participants and Study Design

Through social media advertisement, 38 participants of both sexes volunteered to participate in the present study. All enrolled participants were fully informed about the procedures and risks involved, before a written consent was signed. All participants reported to the laboratory a week prior to the onset of the experimental session week. The exclusion criteria were as follows: arterial hypertension (≥140/90 mmHg), obesity (BMI ≥ 30 kg/m^2^), sedentary lifestyle, previous history of cardiovascular or peripheral arterial disease, previous history of neuromuscular injuries, smoking habits, supplement, drug medication, and contraceptive pill usage. Based on these criteria, six candidates were excluded from participation, while two participants dropped from the study due to reasons unrelated to the experiments. Thus, 30 healthy, active participants of both sexes completed all the study procedures. This study followed the principles of the Declaration of Helsinki and was approved by the Republic of Slovenia National Medical Ethics Committee (120-487/2018/21). The present study was registered at ClinicalTrials.gov under NCT03690258 identifier on September 26, 2018.

In this repeated-measures randomized controlled trial, the participants were instructed to visit the lab on five separate occasions, including two preliminary visits and three experimental sessions. During both preliminary visits, the participants were advised to refrain from vigorous exercise and caffeine or alcohol consumption 24 h prior to each experimental session. All female participants were tested in the early or mid-follicular phase of their menstrual cycle to minimize the potential effects of hormonal fluctuations on the hemodynamics, blood vessel compliance, and autonomous nervous system regulation (Sims and Heather, 2018). During the first screening visit, their medical histories were taken, and their resting electrocardiograph (ECG), BP, and oxygen saturation were measured (Dash 2000, GE, Milwaukee, United States). The PAR-Q questionnaire was used to obtain data on the exercise safety of the participants involved. On their second visit, participants were familiarized with the laboratory equipment and testing procedures, and their maximal oxygen uptake (V̇O_2_ max.) was determined. Also, all participants took part in one familiarization session of squat exercise on the flywheel ergometer in order to avoid any learning effects throughout. The performance of the Valsalva maneuver during experimental data collection was not allowed. After the completion of the preliminary sessions, the participants were randomly assigned into three different experimental conditions, that is, the flywheel-squat ergometer set at three different moments of inertia (or loads). Each participant served as his/her own control.

**FIGURE 1:**
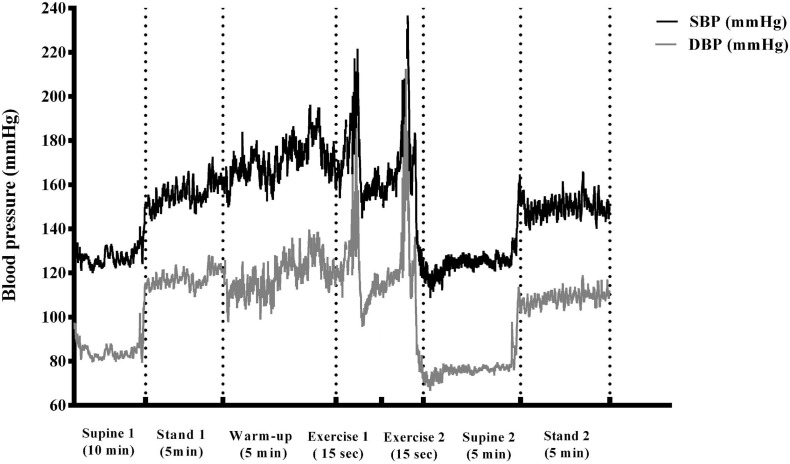
A schematic illustration of the study protocol shown as a typical blood pressure trace recorded throughout the exercise intervention from one participant. Briefly, different phases of the experimental protocol are depicted (*supine 1-baseline, stand 1, stepping warm-up, mounting, exercise 1, rest, exercise 2, supine 2, stand 2*).

Throughout the experimental sessions, all participants arrived to the laboratory at the same time of the day, and rested quietly for 10 min while being instrumented with the Task force monitor (TFM) electrodes, EMG electrodes, and electronic-goniometer. BP was constantly monitored via photoplethysmography, using a pneumatic cuff, as previously described by Blaber et al. (2013). More precisely, the hand was inserted into a neoprene shoulder arm sling to provide additional support and secure the positioning at the level of the heart throughout the exercise. Each experimental procedure typically lasted ∼60 min. Following instrumentation, the recording of the experimental session started. Briefly, each experimental session can be divided into seven different phases during which the participant was instructed to: (i) rest quietly in a supine position for 5 min; (ii) stand for 5 min; (iii) complete a 5 min warm-up stepping protocol (modified Harvard step-test) with a metronome set at 95 beats per minute; (iv) mount on the flywheel ergometer in a standing position; (v) perform two consecutive vigorous, maximal effort squat exercises on the flywheel ergometer (with 2 min of rest in-between following the previously established criteria by Lundberg et al. (2019); ^(^vi) return to supine position for 5 min following the exercise intervention completion; and (vii) stand for 5 min. These time-points were selected to promote the internal validity of the experimental protocol. Each participant completed all three experimental loads within one week, while each experimental procedure was separated by at least 48 h. The experimental protocol is depicted in Figure 1 with full details of the total duration of the protocol explained in the legend of Figure 1. All assessments were performed at a similar time of the day (between 7:30–11:30 a.m., with matched measurement times for each person individually), in a closed and ventilated facility with a temperature range of 20–22°C, by the same researchers, using the same equipment throughout the investigation. All data were collected during February and March to control the effects of seasonal variations in BP regulation (Trozic et al., 2020).

### Experimental Setup and Procedures

#### V̇O_2_ max. Determination

All the participants completed an incremental test on an electronically braked cycle-ergometer (Ergoline 900, Hamburg, Germany) that was synchronized with the Cosmed metabolic cart (CPET, Quark, Rome, Italy). Prior to each cycle-ergometry session, the metabolic analyzer was calibrated against two different gases of known concentrations (room air, and 16.0% O_2_ and 5.0% CO_2_), and the turbine volume transducer was calibrated using a 3-L syringe (model 5530, Hans Rudolph inc., KC, United States) according to the recommendations of the manufacturer. The protocol began with a 3-min warm-up set at 20 W and pedaling frequency of 70 rpm visually displayed throughout the test. The power output was incrementally increased by 20 W each minute (1 W/3 s), until voluntary exhaustion was reached and/or pedaling cadence fell by >5 rpm, despite a strong verbal encouragement from the research staff. Following the test cessation, each participant continued the unloaded pedaling for 5 min to cool-down. Gas exchanges measurements and ventilatory flow were assessed on a breath-by-breath basis via the metabolic cart, and the HR was continuously monitored via a Garmin device (HRM-3 SS, Kansas, United States). The V̇O_2_ max. was accepted as the highest 20 s. V̇O_2_ determined via the rolling average readings sampled during the last minute of the testing, while the peak power output and HR were defined as readings attained at the test cessation, as recently reported (Zubac et al., 2021b).

#### Flywheel Exercise

The exercise intervention(s) were performed on a commercially available flywheel ergometer (nHance, Barcelona, Spain) in an upright position, with both legs positioned on the flywheel-squat ergometer set to low, moderate, or high moments of inertia, corresponding to 0.025, 0.05, and 0.075 kg⋅m^2^, respectively. After the 5-min warm-up stepping protocol, all participants were mounted to the ergometer with their knee joints fully extended in the starting position. In agreement with the manufacturer’s guidelines, the straps were placed around the chest and attached to the ergometer. Following the initial, sub-maximal squat to start the wheel acceleration, the participants performed two sets of seven repetitions of vigorous squat exercises on the flywheel ergometer with maximal effort (with 2 min of rest in-between exercise sets), as previously described in detail (Lundberg et al., 2019).

### Friction Encoder Data Analysis

The average and peak concentric powers for each repetition were sampled at 1 kHz via the Chronopic friction encoder (Chronojump, Barcelona, Spain). The friction encoder and its sensors were positioned on the ergometer as recently reported by Illera-Domínguez et al. (2018). Visual feedback of the power output was displayed to the researchers on a computer screen during the entire experimental protocol via the Chronopic software. Lastly, a self-reported rate of perceived exertion scale (RPE, 1–10) was administered following each experimental session.

### Surface EMG

To assess the EMG activity of the vastus lateralis (VL), a large postural muscle predominantly active during squat exercise throughout the experimental procedures, the SENIAM guidelines for a non-invasive EMG were strictly followed (Hermens et al., 2000). Briefly, the skin was shaved, slightly abraded using NuPrep gel (Weaver and co., Aurora, United States), and cleaned with alcohol to achieve the targeted skin impedance (Z < 10 Ω). EMG recording electrodes (Ambu BlueSensor N, Ballerup, Denmark) were placed over the muscle belly of the left VL along the fiber orientation. Inter-electrode distance was set on 20 mm (measured from center to center of two electrodes), while the reference electrode was placed on the lateral malleolus of the same leg. The EMG signals were amplified via the Noraxon EMG device (TeleMyo 2400T, Noraxon, United States) within a bandwidth frequency (range from 10 to 500 Hz, common mode rejection > 100 dB, gain = 1,500). The knee joint angular displacement was measured via electro-goniometry (model 308, Noraxon, United States) that was secured around the knee joint via micropore tape. The EMG data were sampled at 2 kHz frequency throughout both sets of the flywheel squat exercises. Throughout the entire range of motions, the EMG recordings from the VL muscle were synchronized and triggered with the electro-goniometry data. All signals were filtered and processed via the commercially available Noraxon software. Briefly, the EMG_RMS_ amplitude was calculated from a raw EMG signal over a 100 ms time window during maximal power production of each contraction, and the readings were averaged for further analysis.

### Cardiovascular Response

To assess the BP response, a finger photoplethysmography and a built-in upper arm sphygmomanometer were used. At the start of instrumentation, the participant sat down while a finger plethysmograph cuff was placed on the middle finger on the right arm at the level of the heart. The upper arm sphygmomanometer was positioned on the upper left arm. The cardio-impedance electrodes were positioned according to the manufacturer’s guidelines (TFM, CNSystems, Graz, Austria) to allow a non-invasive insight into the cardiovascular response throughout the experimental protocol. More precisely, the stroke volume and cardiac output were continuously monitored and normalized by the body surface area to obtain the participant-specific parameters, stroke index (SI) and cardiac index (CI) (Trozic et al., 2020). In parallel, the HR was obtained from the bipolar 3-lead electrocardiogram (ECG), placed according to the previously established guidelines for TFM instrumentation (Lackner et al., 2010; Trozic et al., 2020). The power components of the RR interval (RRi) were defined as the time-interval between the two R peaks in the ECG.

### TFM-Derived Data Processing and Analysis

The data were visually inspected for ectopic and aberrant beats, exported to an excel sheet and averaged into specific time points (10 s epochs) to characterize each different phase of the flywheel experimental procedure relevant for the statistical analysis: baseline (epoch 1 = 290–300 s); stand 1 (epoch 2 = 290–300 s); stepping warm-up (epoch 3 = 290–300 s); mounting (epoch 4 = 60–70 s); first set of exercise (epoch 5 = last 10 s); rest (epoch 6 = 60–70 s); second set of exercise (epoch 7 = last 10 s); supine 2 (epoch 8 = 290–300 s); stand 2 (epoch 9 = 290–300 s). Also, epochs representing the maximal cardiovascular response were derived from the abovementioned 10-s epoch and averaged over three maximal heart beats, as previously reported (Ivancev et al., 2007; Trozic et al., 2020). These data were then separately analyzed via the statistical package.

### Statistics

Statistical software was used for all calculations (SPSS 19.0., IBM, Chicago, United States). Normality was confirmed using the Shapiro-Wilk test, with an additional Q-Q plot visual inspection. The 10-s epochs (average data) and maximal readings (three maximal beats) of all cardiovascular data were selected as relevant data points to perform a separate analysis for different phases of the experimental protocols (baseline, stand 1, stepping warm-up, mounting, exercise 1, rest, exercise 2, supine 2, stand 2). The time-course changes of all primary outcomes (e.g., cardiovascular parameters) were entered into a linear mixed model. In this model, the participants were depicted as a random factor, while the time course changes and moments of inertia (low, moderate, and high load) were fixed factors. All neuromuscular data were entered into a mixed general linear model, considering the exercise interventions (the first and the second set of exercise) and experimental sessions (low, moderate, and high loads of inertia) as within factors, with Greenhouse-Geisser correction applied if necessary. Where a significant F-test was identified, a Bonferroni *post hoc* was applied to determine multiple comparisons. For non-parametric data (rate of perceived exertion scale), a Friedman-ANOVA test was applied, followed by a Sign-test separately for each scale. Pearson’s correlation coefficients were calculated to establish associations between percent change in MAP during two consecutive bouts of flywheel exercise for both the average and maximal readings versus the chronological age of the participants included. All data are presented as mean ± SE, and the statistical significance was accepted at *p*-values <0.050.

## Results

### Baseline Measurements

Baseline study population characteristics are given in Table 1. Briefly, 30 participants of both sexes (37% women) completed all the study procedures. According to the cycle-ergometry data, all participants had attained their maximal age-predicted HR during exercise, while both the secondary criteria, including V̇_E_ peak, and the respiratory exchange ratio readings indicate that the limits to the power output were reached. There were no age-differences observed between the men and women included in this study.

**TABLE 1:** Participants characteristics.

***N* (%, women)**	**30 (37%)**
**Anthropometric characteristics**	
Age, y	33 ± 4
Body height, cm	176 ± 2
Body mass, kg	78 ± 3
Body mass index, kg⋅m^–2^	24 ± 1
**Cardiovascular parameters**	
Resting HR, bpm	65 ± 2
Resting SBP, mmHg	118 ± 2
Resting DBP, mmHg	67 ± 2
Resting MAP, mmHg	84 ± 1
Resting SaO_2_, %	98 ± 1
**Cardiorespiratory fitness**	
V̇_E_ peak, L⋅min^–1^	121 ± 6
V̇O_2_ max., L⋅min^–1^	3.2 ± 0.2
V̇O_2_ max., mL⋅kg⋅min^–1^	41 ± 2
RER,	1.22 ± .02
HR max., bpm	180 ± 2
PPO, W	250 ± 12

*Abbreviations: HR, heart rate; SBP, systolic blood pressure; DBP, diastolic blood pressure; MAP, mean arterial pressure; SaO_2_, oxygen saturation; V̇_*E*_, peak pulmonary ventilation, V̇O_2_ max., maximal oxygen uptake, RER, respiratory exchange ratio, PPO, peak power output. Data are given as mean ± SE.*

### Cardiovascular Results

The average and maximal values of the cardiovascular response to flywheel loading are given in Table 2. For the average BP readings across the three different flywheel exercise interventions, interaction effects (load vs time) were observed for the MAP and diastolic blood pressure (DBP) (*p* = 0.031 and *p* = 0.005, respectively). A *post hoc* test revealed an increment in the MAP and DBP when compared to the resting (baseline) values, during both first and second sets of the exercise bouts (*p* = 0.001). There was no interaction effect (load vs time) for the systolic blood pressure (SBP, *p* = 0.168). However, time and load effects were observed for the SBP during the exercise bouts when compared to the baseline (*p* = 0.001, respectively), where *post hoc* revealed that the SBP increased from the baseline values of 115 ± 3 mmHg (across all three experimental sessions) by 57, 64, and 67%, proportional to the moment of inertia increment, respectively. No interactions were observed for the HR (*p* = 0.268), indicating comparable readings across all three flywheel loadings. However, an increase in HR was observed during all flywheel loadings compared to the baseline (time effect: *p* = 0.001), whereas the HR at a high level of flywheel load was 10 bpm higher (load effect: *p* = 0.001) compared to readings at low and moderate loadings.

**TABLE 2:** Average readings of the cardiovascular response to different levels of the flywheel exercise.

	**Supine 1**	**Stand 1**	**Warm-up**	**Mounting**	**Exercise 1**	**Rest**	**Exercise 2**	**Supine 2**	**Stand 2**	***Sig.***
**SBP (mmHg)**										
Low	114 ± 4	133 ± 4^$^	153 ± 4^$^	147 ± 4	171 ± 4^$^	148 ± 4	179 ± 3^$^	116 ± 4	131 ± 4	*–*
Moderate	118 ± 4	137 ± 4^$^	159 ± 4^$^	153 ± 4	188 ± 4^$#^	152 ± 4	187 ± 4^$#^	119 ± 4	138 ± 4	*–*
High	114 ± 4	137 ± 4^$^	158 ± 4^$^	151 ± 4	190 ± 4^$#^	150 ± 4	198 ± 5^$#^	119 ± 5	136 ± 5	*p* < 0.05
**DBP (mmHg)**										
Low	73 ± 3	95 ± 3^$^	94 ± 3	92 ± 3	117 ± 3*^$^	93 ± 3	120 ± 3*^$^	67 ± 3	91 ± 3	*–*
Moderate	75 ± 3	98 ± 3^$^	101 ± 3	98 ± 3	134 ± 4*^$#^	100 ± 4	130 ± 3^$#^	68 ± 3	99 ± 3	*–*
High	73 ± 3	99 ± 3^$^	97 ± 3	96 ± 3	135 ± 3*^#^	97 ± 3	137 ± 3*^#^	66 ± 3	95 ± 3	*p* < 0.05
**MAP (mmHg)**										
Low	88 ± 3	109 ± 3^$^	116 ± 3^$^	112 ± 3	142 ± 4*^$^	114 ± 4	146 ± 4*^$^	85 ± 4	106 ± 3	*–*
Moderate	92 ± 4	112 ± 4^$^	122 ± 3^$^	118 ± 4	157 ± 6*^$#^	120 ± 4	154 ± 4^$#^	87 ± 4	113 ± 4	*–*
High	89 ± 3	113 ± 3^$^	120 ± 4^$^	115 ± 3	158 ± 3*^$#^	117 ± 3	162 ± 4*^$#^	86 ± 3	111 ± 3	*p* < 0.05
**HR (bpm)**										
Low	66 ± 3	82 ± 3^$^	116 ± 3^$^	114 ± 3	128 ± 3^$^	108 ± 3	128 ± 3^$^	77 ± 3	92 ± 3	*–*
Moderate	68 ± 3	83 ± 2^$^	119 ± 3^$^	117 ± 3	136 ± 3^$#^	108 ± 3	137 ± 2^$#^	79 ± 3	93 ± 3	*–*
High	69 ± 3	82 ± 3^$^	118 ± 3^$^	117 ± 3	138 ± 3^$#^	113 ± 3	137 ± 3^$#^	77 ± 3	94 ± 3	*p* < 0.05
**CI (L⋅min⋅m^2^)**										
Low	3.2 ± 0.2	2.8 ± 0.2	5.8 ± 0.2^$^	4.9 ± 0.2	6.3 ± 0.2^$^	4.0 ± 0.2	6.1 ± 0.2^$^	4.1 ± 0.2	2.9 ± 0.2	*–*
Moderate	3.5 ± 0.2	2.9 ± 0.2	6.0 ± 0.2^$^	5.1 ± 0.2	5.9 ± 0.2^$^	4.2 ± 0.2	6.1 ± 0.2^$^	4.3 ± 0.2	3.1 ± 0.2	*–*
High	3.4 ± 0.2	2.9 ± 0.2	6.0 ± 0.2^$^	4.9 ± 0.2	5.8 ± 0.2^$^	4.0 ± 0.2	5.7 ± 0.2^$^	4.2 ± 0.2	3.1 ± 0.2	*p* < 0.05
**SI (mL⋅m^2^)**										
Low	50.0 ± 1.7	34.2 ± 1.7	49.7 ± 1.7	43.1 ± 1.7	48.6 ± 1.7	37.7 ± 1.7	47.3 ± 1.7	54.6 ± 1.7	32.7 ± 1.7	*–*
Moderate	50.9 ± 1.8	35.1 ± 1.7	51.3 ± 1.7	44.2 ± 1.7	43.9 ± 1.8^#^	39.5 ± 1.7	44.5 ± 1.7	55.1 ± 1.7	33.9 ± 1.7	*–*
High	49.6 ± 1.7	34.9 ± 1.7	50.6 ± 1.7	43.0 ± 1.7	42.6 ± 1.7^#^	36.8 ± 1.7	41.6 ± 1.8^#^	55.4 ± 1.7	33.2 ± 1.7	*P* < 0.05
**TPRI (dyne⋅s m^2^ cm^^5^)**										
Low	2277 ± 99	3198 ± 99^$^	1659 ± 99	1853 ± 101	1869 ± 99	2329 ± 100	1955 ± 100	1694 ± 101	2846 ± 100	*–*
Moderate	2195 ± 102	3169 ± 102^$^	1655 ± 102	1849 ± 102	2215 ± 103^$^	2354 ± 102	2083 ± 101	1664 ± 101	2962 ± 102	*–*
High	2197 ± 99	3245 ± 99^$^	1651 ± 99	1883 ± 100	2260 ± 99^#[*dollar*]^	2354 ± 99	2394 ± 99^#^	1665 ± 99	2946 ± 99	*P* < 0.05

Abbreviations: SBP, systolic blood pressure; DBP, diastolic blood pressure; MAP, mean arterial pressure; HR, heart rate; CI, cardiac index; SI, stroke volume index; TPRI, total peripheral resistance index; Sig., significance level. Data are given as mean ± SE. *Significant interaction effect between supine 1 (baseline) and exercise 1 and exercise 2 across three different levels of iso-inerial loading. #Significant load effect - different from low-level iso-inertial load. $Significant time effect between supine (baseline) and exercise 1 and exercise 2.

For the maximal readings of the cardiovascular response to flywheel load, an interaction effect was observed for all BP response variables, Table 3. The rise in the SBP was proportional to the flywheel load increment (interaction effect: *p* = 0.001), with the SBP reaching the highest values (by 214 ± 4 mmHg, *p* = 0.001) during a high flywheel load. The DBP and MAP readings did follow the same above-mentioned pattern, with the highest MAP readings (179 ± 4 mmHg) reached during the highest flywheel load for both exercise bouts (*p* = 0.001). No interaction was observed for the maximal HR adjustments (*p* = 0.424), as the HR to different exercise interventions was comparable across the three flywheel loads. However, an increase in the HR, compared to the baseline, was observed across all three loads during both exercise bouts (time effect: *p* = 0.001), while the greatest increase in the HR was observed during a moderate load, with the HR reaching up to 141 bpm.

**TABLE 3:** Maximal readings of the cardiovascular response to different levels of the flywheel exercise.

	**Supine 1**	**Stand 1**	**Warm-up**	**Mounting**	**Exercise 1**	**Rest**	**Exercise 2**	**Supine 2**	**Stand 2**	***Sig.***
**SBP (mmHg)**										
Low	114 ± 4	134 ± 4^$^	156 ± 4^$^	148 ± 4	184 ± 4*^$^	148 ± 4	191 ± 4*^$^	117 ± 4	130 ± 4	**–**
Moderate	118 ± 4	137 ± 4^$^	159 ± 4^$^	154 ± 5	203 ± 4*^$#^	153 ± 4	200 ± 4^$#^	119 ± 4	138 ± 4	**–**
High	115 ± 4	138 ± 4^$^	158 ± 4^$^	152 ± 4	201 ± 4*^$#^	150 ± 4	214 ± 4*^$#^	120 ± 4	137 ± 4	*P* < .005
**DBP (mmHg)**										
Low	72 ± 3	95 ± 3^$^	94 ± 3	92 ± 3	125 ± 4*^$^	94 ± 4	131 ± 4*^$^	67 ± 3	91 ± 3	**–**
Moderate	76 ± 3	99 ± 3^$^	102 ± 4	99 ± 3	149 ± 4*^$#^	101 ± 3	142 ± 3^$#^	69 ± 3	99 ± 3	**–**
High	74 ± 3	99 ± 4^$^	98 ± 4	96 ± 4	144 ± 4*^$#^	98 ± 3	153 ± 4*^$#^	66 ± 3	96 ± 3	*P* < 0.05
**MAP (mmHg)**										
Low	98 ± 4	110 ± 4^$^	117 ± 4^$^	112 ± 4	153 ± 4*^$^	114 ± 4	157 ± 4*^$^	87 ± 4	106 ± 3	**–**
Moderate	92 ± 4	113 ± 4^$^	123 ± 4^$^	119 ± 4	174 ± 4*^$#^	119 ± 4	168 ± 4^$#^	87 ± 3	114 ± 4	**–**
High	90 ± 4	113 ± 4^$^	120 ± 4^$^	116 ± 4	169 ± 4*^$#^	118 ± 4	179 ± 4*^$#^	87 ± 4	112 ± 4	*P* < 0.05
**HR (bpm)**										
Low	65 ± 3	82 ± 3^$^	115 ± 3^$^	114 ± 3	133 ± 3^$^	107 ± 3	132 ± 3^$^	77 ± 3	91 ± 3	**–**
Moderate	68 ± 3	82 ± 3^$^	119 ± 3^$^	117 ± 3	139 ± 3^$^	107 ± 3	141 ± 3^$#^	78 ± 3	92 ± 3	**–**
High	69 ± 3	82 ± 3^$^	117 ± 3^$^	116 ± 3	137 ± 3^$^	113 ± 3	138 ± 3^$#^	77 ± 3	94 ± 3	*P* < 0.05
**CI (L⋅min⋅m^2^)**										
Low	3.2 ± 0.2	2.8 ± 0.2	5.7 ± 0.2^$^	4.8 ± 0.2	6.6 ± 0.2*^$^	3.9 ± 0.2	6.3 ± 0.2*^$^	4.1 ± 0.2	2.9 ± 0.2	**–**
Moderate	3.5 ± 0.2	2.9 ± 0.2	5.9 ± 0.2^$^	5.1 ± 0.2	5.9 ± 0.2*^$^	4.1 ± 0.2	6.5 ± 0.2*^$^	4.2 ± 0.2	3.1 ± 0.2	**–**
High	3.4 ± 0.2	2.8 ± 0.2	5.8 ± 0.2^$^	4.9 ± 0.2	5.8 ± 0.2*^$^	4.0 ± 0.2	5.6 ± 0.2^$^	4.2 ± 0.2	3.1 ± 0.2	*P* < 0.05
**SI (mL⋅m^2^)**										
Low	49.8 ± 1.7	33.9 ± 1.8	49.5 ± 1.8	42.9 ± 1.8	49.1 ± 1.8	39.5 ± 1.7	47.6 ± 1.8	54.4 ± 1.8	32.0 ± 1.7	**–**
Moderate	50.7 ± 1.8	34.9 ± 1.8	51.1 ± 1.8	43.9 ± 1.8	44.1 ± 1.8^#^	39.3 ± 1.8	45.8 ± 1.8	54.9 ± 1.8	33.7 ± 1.8	**–**
High	49.4 ± 1.8	34.8 ± 1.8	50.4 ± 1.8	42.8 ± 1.7	42.7 ± 1.7^#^	36.5 ± 1.8	41.3 ± 1.8^#^	55.2 ± 1.8	33.1 ± 1.8	*P* < 0.05
**TPR (dyne⋅s⋅m^2^⋅cm^5^)**										
Low	2290 ± 104	3211 ± 104^$^	1673 ± 104	1867 ± 106	1946 ± 106	2343 ± 105	2066 ± 106	1708 ± 105	2860 ± 105	**–**
Moderate	2209 ± 107	3183 ± 106^$^	1669 ± 104	1862 ± 106	2404 ± 110*	2368 ± 107	2168 ± 107	1678 ± 107	2976 ± 107	**–**
High	2211 ± 104	3259 ± 104^$^	1665 ± 104	1897 ± 104	2442 ± 105*	2368 ± 104	2705 ± 106*	1679 ± 104	2959 ± 104	*P* < 0.05

Abbreviations: SBP, systolic blood pressure; DBP, diastolic blood pressure; MAP, mean arterial pressure; HR – heart rate; CI, cardiac index; SI, stroke volume index; TPRI, total peripheral resistance index. Sig., significance level. Data are given as mean ± SE. *Significant interaction effect between supine 1 (baseline) and exercise 1 and exercise 2 across three different levels of iso-inertial loading. #Significant load effect - different from low-level iso-inertial load. $Significant time effect between supine (baseline) and exercise 1 and exercise 2.

All the normalized cardiovascular parameters (average values) had a similar response to different flywheel loads, and no interaction was depicted for the SI, CI, and total peripheral resistance index (TPRI) (*p* = 0.160, *p* = 0.293, *p* = 0.065, respectively). However, a load-dependent drop in SI during flywheel exercise was observed (*p* = 0.001), while in parallel, a two-fold greater increase in CI and TPRI throughout the exercise bouts was documented when compared to rest (time effect: *p* = 0.001). The TPRI increased by 4 and 10% (average readings) during the highest level of flywheel loading (compared to low and moderate loading) during both exercise bouts (*p* = 0.001), respectively. For maximal values, an interaction was observed for both the normalized CI (*p* = 0.020) and TPRI (*p* = 0.001), but not for the SI (*p* = 0.122). A *post hoc* test revealed comparable increases in the CI across all three loads (*p* = 0.001), while a SI decline was flywheel load-dependent (*p* = 0.001). The TPRI increased by 11 and 22% (*p* = 0.001, for both exercise bouts, respectively) during the highest load (compared to the low and moderate flywheel loads).

No interaction was observed for the relative changes in the mean arterial pressure (MAP%) across the three flywheel loadings and two consecutive exercise bouts (for both the average and maximal readings, *p* = 0.717, and *p* = 0.318, respectively).

**FIGURE 2:**
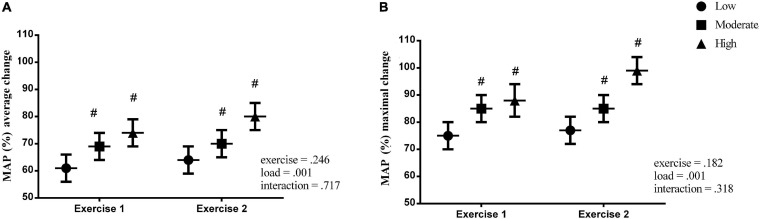
Percent change in the mean arterial pressure (MAP%) during the flywheel exercise for both the average and maximal readings. # – significant load effect – different from low-level iso-inertial load.

However, the rise in MAP (%) was load-dependent (in both the average and maximal readings, *p* = 0.001, respectively), and the data indicate that the increase in MAP (%) during the first exercise bout did not influence the MAP (%) rise throughout the second exercise bout (exercise effect: *p* = 0.246 and *p* = 0.182, respectively, Figure 2).

### Correlations

**FIGURE 3:**
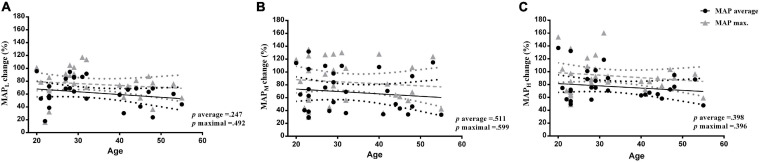
Correlation analysis between the changes in the mean arterial pressure (both average black circles and maximum gray triangles) versus the age of the participants per load (*Low A set* 0.025; *Moderate B* 0.05; *and High C* 0.075 kg m^2^*, moments of inertia*).

Figure 3 depicts the changes in the MAP (both average as black circles and maximum as gray triangles) versus the age of the participants per load (Low: A; Middle: B; High: C). The graphs show that no correlations were observed between the increase in the MAP (%) (for both average and maximal readings) and the age of the participants for (A) low (*r* = −0.225, *p* = 0.247; *r* = −0.134; *p* = 0.492), (B) moderate (*r* = −0.127, *p* = 0.511; *r* = −0.108, *p* = 0.599), and (C) high level (*r* = −0.159; *p* = 0.398; *r* = −0.161; *p* = 0.396) of moment of inertia.

In Table 4, data from the secondary outcome variables are given. The neuromuscular response to the flywheel exercise indicates no load *vs* session interaction effects for the average or peak concentric power (*p* = 0.358; *p* = 0.596, respectively), nor exercise duration (*p* = 0.243). However, load effects (*p* = 0.001) were observed across the above-mentioned variables, where the average and peak power decreased proportionally to the moment of inertia increment. There were no interaction effects observed for the EMG_RMS_ VL activity (*p* = 0.856), and the knee angle flexion pattern was similar across all three experimental conditions and the two exercise bouts within each experiment (*p* = 0.203).

**TABLE 4:** Neuromuscular parameters of the participants.

**Level of inertia (kg⋅m^2^)**	**Low**	**Moderate**	**High**	**Time**	**Load**	**Interaction**
	**0.025**	**0.050**	**0.075**			
Average concentric power_1_, W	901 ± 49	722 ± 49#	643 ± 48#	–	–	–
Average concentric power_2_, W	880 ± 49	751 ± 49#	635 ± 49#	0.998	0.001	0.358
Peak concentric power_1_, W	1581 ± 87	1226 ± 88#	1082 ± 86#	–	–	–
Peak concentric power_2_, W	1547 ± 87	1258 ± 88#	1080 ± 87#	0.976	0.001	0.596
Exercise duration_1_, s	11.3 ± 0.3	13.3 ± 0.4#	14.0 ± 0.3#	–	–	–
Exercise duration_2_, s	11.5 ± 0.3	13.1 ± 0.3#	13.5 ± 0.3#	0.078	0.001	0.243
EMG_1__RMS_ VL, mV	0.352 ± 0.034	0.324 ± 0.034	0.341 ± 0.034	–	–	–
EMG_2 RMS_ VL, mV	0.339 ± 0.034	0.305 ± 0.034	0.299 ± 0.033	0.271	0.464	0.856
Knee angle flexion_1_,	−53.6 ± 3.8	−57.4 ± 3.8	−54.7 ± 3.7	–	–	–
Knee angle flexion_2_,	−53.9 ± 3.8	−52.3 ± 3.9	−57.1 ± 3.8	0.557	0.646	0.203
RPE, 1-10	5 ± 1	6 ± 1	7 ± 1#	–	–	0.001

*Abbreviations: Low, moderate, and high levels of iso-inertial load corresponding to 0.025; 0.05, and 0.075 kg⋅m^2^; 1, first bout of exercise, 2, second bout of exercise. Data are given as mean ± SE. *#Post-hoc* of the load effect – significantly different from low-level iso-inertial load.*

## Discussion

In the present study, we found that a vigorous, maximal effort, whole-body flywheel squat exercise results in acute, exercise-induced rises in BP and HR (Tables 2, 3, for both the average and the maximal readings, respectively). Our findings on the exercise-induced BP responses seem to be to somewhat lower (especially during low and moderate loads, with the average BP readings reaching ∼184/125 and ∼203/144 mmHg), compared to those reported by Lovell et al. (2011). They had outlined that the BP reached as high as ∼231/130 mmHg in ∼70-year-old sedentary men (age range from 70–80 years) during maximally loaded, classical squat-exercises. However, the study is unclear in stating whether the abrupt BP response is due to a poor aerobic fitness or advanced age. Our investigation builds upon previous findings (Lamotte et al., 2010; Massaferri et al., 2015; Trinity et al., 2018) by looking at the cardiovascular response in healthy, active participants of wider age-range and both sexes during an acute resistance exercise. The present findings of the BP and HR response are consistent with our recent work on flywheel exercise and cardiovascular response in men of varying age and cardiorespiratory fitness (Zubac et al., 2021a). In that study, a sharp and transient BP response (230/115 mmHg) to a high level iso-inertial loading (0.075 kg m^2^) was reported. The present work also provides a novel insight into the cardiovascular response via a simultaneous measurement of photoplethysmography, cardio-impedance electrodes, and ECG, and overcomes the methodological pitfalls associated with photoplethysmography-based assessments alone (Lovell et al., 2011; Massaferri et al., 2015; Zubac et al., 2021a). The present findings expand to that and add information on low and moderate loadings in parallel to the EMG activation patterns of the VL muscle. Furthermore, the flywheel exercise model, unlike other whole-body resistance exercise routines, evades certain technical limitations of the non-invasive hemodynamics assessment by allowing the finger-cuff to be positioned at a fixed level of the heart to provide a unique opportunity to study cardiovascular demands during resistance exercise. Thus, as a certain novelty, the data presented here add to the sparse literature on the non-invasive assessment of the cardiovascular responses during a high-intensity resistance exercise (Tables 2, 3), since the previous work of Lovell et al. (2011) and Zubac et al. (2021a) were limited to HR and BP data during exercise, and failed to provide any deeper insight into the complex cardiovascular adjustments during a vigorous whole-body squat exercise.

The central command and peripheral reflex mechanism are considered to be key pathways responsible for sudden adjustments in the cardiovascular dynamics throughout resistance-based exercises (Mitchell, 2013; Fisher et al., 2015). In parallel, due to a large muscle mass activation, the circulatory readjustments were likely needed to meet the metabolic demands at a tissue level (Mueller et al., 2018). In our study, the changes in the CI, SI, TPRI were caused by excessive, upright, whole-body flywheel squat exercises, which include seven robust concentric to eccentric bouts (e.g., a forceful muscle loading via explosive contractions, completed within ∼11–13 s, Table 4). These excessive, lower-limb contractions during flywheel squat exercises were likely instrumental to the large blood vessel occlusion, due to the intramuscular mechanical and intrathoracic pressure development, further leading to an attenuated venous return. Apparently, an inadequate cardiac preload is the most reasonable mechanism to clarify a slight drop in the SI throughout larger loads, followed by an abrupt rise in the HR. In fact, the flywheel exercise mediated a small, but load-specific SI reduction (for moderate and high inertial loads by ∼11%, compared to lower loads), whereas in parallel, a sustainable increase in the HR was observed, especially during the highest loading. This, in turn, compensated for a reduced SI, and maintained the CI during the flywheel exercises. Besides, our participants were instructed to perform the flywheel exercise test as fast as possible, thus, we can only speculate whether the cardiac filling was re-established during the eccentric portion of the squat movement. An excessive rise in the HR was observed during the flywheel exercise (from 65 bpm during rest to ∼135 bpm through the experimental conditions), and was likely mediated by the interplay among peripheral sensory mechanism and sympathetic cardiac activation. However, a direct mechanism governing the load-dependent cardiovascular response to flywheel is rather difficult to explain in detail, primarily due to our non-invasive data collection approach and the complexity of the sympathetic outflow assessment during a vigorous whole-body exercise. The theoretical nature of our explanations seems to corroborate with textbook physiology of Rowell et al. (1996), who indicated that the combined effects of the peripheral sensory mechanisms drive the increased sympathetic activity and are instrumental to the cardiovascular adjustments during a dynamic exercise. Here, we did not directly measure the muscle sympathetic nerve activity following the flywheel exercise, and any discussion is, therefore, rather speculative.

### Blood Pressure Dynamics

Contrary to our primary hypothesis, we observed a robust rise in the MAP (%) during all three flywheel loads, which were not associated with the chronological age of the participants (Figure 3). A positive correlation between the percentage of change in the MAP and the age of these participants were anticipated, as we presumed that the MAP would increase in proportion to the advancing age, even if data on MAP adjustments during a vigorous variable load exercise are scarce. What we do know from previous work had influenced our hypothesis, since a large body of literature suggests that ageing has an adverse effect on the cardiovascular system in humans (Arbab-Zadeh et al., 2004; Seals et al., 2011; Fletcher et al., 2013). Briefly, previous work depicted several profound age-related changes, including left ventricular wall thickening (Shibata et al., 2018), impaired endothelial function (Seals et al., 2011), and greater arterial stiffening (Seals et al., 2011; Shibata et al., 2018). Usually, these profound changes of the cardiovascular system advance and become steeper when turning >45 years in men, and >50 years of age in women (Pescatello et al., 2004). According to the American College of Sports Medicine (ACSM), a lifelong trend in the SBP increment in the general population is observed in longitudinal studies, secondary to progressive arterial stiffening (Pescatello et al., 2004; Shibata et al., 2018). The V̇O_2_ max. is considered to be a vital, clinically relevant, physiological indicator of the general population’s functional capacity (Valenzuela et al., 2020), since oxygen uptake under stress tests integrate multifactorial physiological responses across different systems to provide a global measure of cardiorespiratory fitness. The present findings indicate a substantially higher cardiorespiratory fitness level of our participants, as observed from the average V̇O_2_ max. values reached during the cycle-ergometry test (41 ± 2 mL kg min^–1^), compared to normative reference standards recently published by Kaminsky et al. (2017) in a large epidemiological study published in the United States. Thus, it is quite possible that the greater cardiorespiratory fitness, observed in our healthy adults of varying ages (20–55 years), had attenuated, at least partially, the age-related MAP rise during our flywheel exercise. This notion is supported via recent findings of Trinity et al. (2018) who reported an abrupt rise in BP during a submaximal plantar flexion exercise in aging, sedentary women. Collectively, the data presented here fit well within the healthy ageing research paradigm recently discussed by Harridge and Lazarus (2017), since a long-lasting physical activity involvement apparently protects against a robust rise in MAP during a vigorous exercise.

### Recommendations for Flywheel Exercises

Regarding the exercise testing procedures in adult populations, both the ACSM’s and the AHA’s position strategies (Pescatello et al., 2004; Fletcher et al., 2013) and advise that even middle-aged persons should perform an exercise capacity testing prior to engaging in a vigorous exercise program. Their policies suggest, as a hazardous hypertensive response to stress-induced exercise, a threshold of BP rise of >250/115 mmHg during testing (typically observed during the Bruce test), and they conclude that exercise should be terminated when the above-mentioned values are reached, primarily due to safety reasons. In the present study, the average BP response during the flywheel exercise were ∼200/130 mmHg, and these values are slightly lower than those reported in similar studies (Lovell et al., 2011; Massaferri et al., 2015). The current data are noteworthy, since previous work on continuous BP measurements was focused exclusively on the incremental testing, as recently proposed by Miyai et al. (2021), whereas less is known regarding the optimal BP response to resistance exercise muscle loading. In fact, additional research is warranted, since there is no consensus yet concerning the criteria for a hypertensive response threshold in resistance-based exercises for different populations including sedentary adults, moderately fit adults, or seniors. Although the present study provides a novel insight into the acute cardiovascular response to the whole-body flywheel exercise, our findings are limited by a few essential factors. In particular, the most important limitation is that we were unable to recruit healthy, active participants older than > 55 years of age. Also, no senior athletes were recruited for this study. If that would be the case, the study would allow for a better matching of the participants’ age and physical activity level. Currently, this limits our interpretation of the observed differences, being due to age or physical inactivity. However, this was a compromise we took to control for the internal and external validity of the data collected, since the age-related differences between sexes (including menopause) dictate the physiological response to exercise. Therefore, a major recommendation for future studies is to address these issues, especially among the active senior population.

In conclusion, this was the first study to quantify the magnitude of the BP response to the flywheel exercise in healthy adult humans of varying age. Apparently, exercise prescription at larger loads of inertia during the flywheel exercise seem to impose a substantial burden to the cardiovascular system, although the study population’s age-related differences did not modulate a robust relative rise in the MAP during the flywheel loads. Thus, while countering muscle atrophy with a high-load exercise, as a prerequisite of flywheel exercise safety optimization, exercise-scientists should focus on quantifying the magnitude of the cardiovascular response during specifically-tailored, personalized flywheel exercises. Taken together, to ensure that exercise interventions can be safely prescribed for different populations, including seniors, future studies should examine in more detail the interplay between muscle loads at different intensities and the subsequent BP response during exercise and recovery.

## Data Availability Statement

The original contributions presented in the study are included in the article/Supplementary Material, further inquiries can be directed to the corresponding author/s.

## Ethics Statement

The studies involving human participants were reviewed and approved by this study was approved by the Republic of Slovenia National Medical Ethics Committee (120-487/2018/21). The patients/participants provided their written informed consent to participate in this study.

## Author Contributions

DZ, VI, ZV, CM, and IT performed material preparation, data collection, and analysis. DZ, NG, BŠ, CM, and RP wrote the first draft of the manuscript. All authors commented on previous versions of the manuscript, contributed to the study conception and design, and read and approved the final manuscript.

## Conflict of Interest

The authors declare that the research was conducted in the absence of any commercial or financial relationships that could be construed as a potential conflict of interest.
