# Supplementary material for: A Randomized Crossover Trial on the Acute Cardiovascular Demands During Flywheel Exercise
Source: Front Physiol. 2021 Jun 25;12:665462. doi: 10.3389/fphys.2021.665462 (PMC8267577; doi:10.3389/fphys.2021.665462)
Supplement: Supplementary file 1 [file Data_Sheet_1.PDF]

MODIFIED PAR-Q FORM

Name ____

AGE: ___/____/____ Phone (h): _____________ Phone(w):____________

1) Has a doctor ever said you have a heart condition and you should only do physical activity recommended by a doctor? **Yes / No**

2) When you do physical activity, do you feel pain in your chest? **Yes / No**

3) When you were not doing physical activity, have you had chest pain in the past month? **Yes / No**

4) Do you ever lose consciousness or do you lose your balance because of dizziness? **Yes/ No**

5) Do you have **any** muscle, joint or bone problem? **Yes / No**

6) Have you ever suspected or been told that you have a muscle, joint or bone problem, or have you experienced undue pain in your lower limbs during the last year? (This might include, for example, 'shin splints' or tendonopathies.) **Yes /No**

7) Are you currently taking any medications? **Yes / No**

8) Do you have insulin dependent diabetes? **Yes / No**

9) Have you ever had heat stroke or heat intolerance? **Yes / No**

10) Do you have kidney or gut dysfunction? **Yes / No**

11) Do you know of any other reason you should not exercise? **Yes / No**

Your signature: _____________________________Date: __/___/___
